# Localization diagnosis of low back pain in a population-based study of a Japanese mountain village

**DOI:** 10.1371/journal.pone.0282115

**Published:** 2023-02-23

**Authors:** Norihiko Takegami, Koji Akeda, Junichi Yamada, Tatsuhiko Fujiwara, Akinobu Nishimura, Akihiro Sudo

**Affiliations:** Department of Orthopaedic Surgery, Mie University Graduate School of Medicine, Tsu City, Mie, Japan; University of Toronto, CANADA

## Abstract

**Purpose:**

The purposes of this study were to investigate 1) the location of low back pain (LBP) and 2) the relationships between the location of LBP and the LBP intensity or the quality of life (QoL) in a population-based study.

**Methods:**

The location of LBP was categorized into four areas using palpation: midline of the lumbar region, paravertebral muscles, upper buttock, and sacroiliac joint. The extent of LBP in the situations/positions was assessed. The relationships between the location of LBP and the extent of LBP on the QoL were statistically analyzed.

**Results:**

174 participants (average age: 72.3 years-old) were analyzed in this study. 93 participants (53.4% of the total) who had experienced LBP in the past three months were included in the LBP-positive group. Numerical rating scale (NRS) scores of the LBP-positive group were highest in the standing position. 51.6% of the LBP-positive group had LBP at the midline of the lumbar region, 40.9% at the paravertebral muscles, 28.0% at the upper buttock, and 15.1% at the sacroiliac joint. In the standing position, NRS scores of LBP at the upper buttock were significantly higher than those at the midline of the lumbar region and the paravertebral muscles (P<0.05). The Oswestry Disability Index scores of participants who had pain at the upper buttock were significantly higher than those at the midline of the lumbar region or paravertebral muscles (P<0.05).

**Conclusion:**

Our study was the first report to investigate the location of LBP using palpation in a population-based study. LBP localization was significantly associated with LBP intensity and LBP-related QoL.

## Introduction

The cause of low back pain (LBP) was previously unclear in about 85% of patients; such cases are referred to as ‘non-specific LBP’ [1]. Recent detailed examinations, questioning, and image diagnosis of LBP patients by orthopedists allowed the identification of 78% of specific LBP cases [2].

Multifactorial causes that include biological, psychological, and social factors contribute to pathogenesis of LBP [3]. Furthermore, the anatomical structures that constitute the lumbar spine (intervertebral discs, facet joints, sacroiliac joints, and soft tissues) can be the cause of pain stressors, and each of these structures, alone or in combination, can contribute to LBP. However, image analyses, including radiography, computed tomography (CT) and/or magnetic resonance imaging (MRI), rarely identify the anatomical pathologies of LBP [4]. It is of great importance to the diagnose LBP, not only by image assessment but also by physical examination including palpation of lumbar anatomical structures, to identify the pain location in the clinical setting [5]. Previous studies reported that the palpation method was useful for the pain provocation in diagnosing LBP [6,7]. However there has been no population-based study investigating the location and intensity of LBP in the general population.

The purposes of this study were to examine 1) the location of LBP in a medical examination of common inhabitants, and 2) the relationships between the location of LBP and the extent of LBP on the quality of life (QoL) in a population-based study of a Japanese mountain village.

## Methods

### Participants

This study was conducted with the approval of the Clinical Research Ethics Review Committee of Mie University Hospital, and all participants provided written informed consent before enrollment in the study. Data were obtained from November 25 to December 15 in 2017 from participants in the Miyagawa study. The Miyagawa study started in 1997 and is a population-based cohort study conducted to identify factors associated with knee osteoarthritis, osteoporosis, vertebral fracture, disc degeneration, and locomotive syndrome by collecting data from a representative sample of a local elderly Japanese population [8–11]. Participants aged more than fifty years-old were recruited from inhabitants of Miyagawa (Odai-cho), a mountain village located in the center of Mie Prefecture, Japan.

### Clinical interview

Participants completed an interviewer-administrated questionnaire that included information on age, gender, the presence of LBP, the presence of comorbidities, and the evaluation of QoL. Comorbidities included hypertension, diabetes mellitus, dementia, Parkinsonism, cerebrovascular disorder, ischemic heart disease, and smoking. Anthropometric measurements included body height, body weight, body mass index (BMI), and bone mineral density (BMD). The BMD of the forearm was measured using dual-energy x-ray absorptiometry. (DCS-600EXV, Aloka, Tokyo). All participants were interviewed by experienced orthopedists about the presence of LBP. Low back pain was defined as pain in the area on the posterior aspect of the body from the lower margin of the twelfth ribs to the lower gluteal folds [12]. They were asked ‘Do you have low back pain within the recent three months?’ Participants who answered “yes” were defined as the LBP-positive group.

### Evaluation of LBP intensity

LBP intensity was measured on an 11-point pain intensity numerical rating scale (NRS) [13,14]. The extent of LBP in the following situations/positions was assessed using the NRS: 1. morning awakening; 2. walking; 3. standing; 4. sitting; and 5. lying down.

### Physical examination

**Fig 1:**
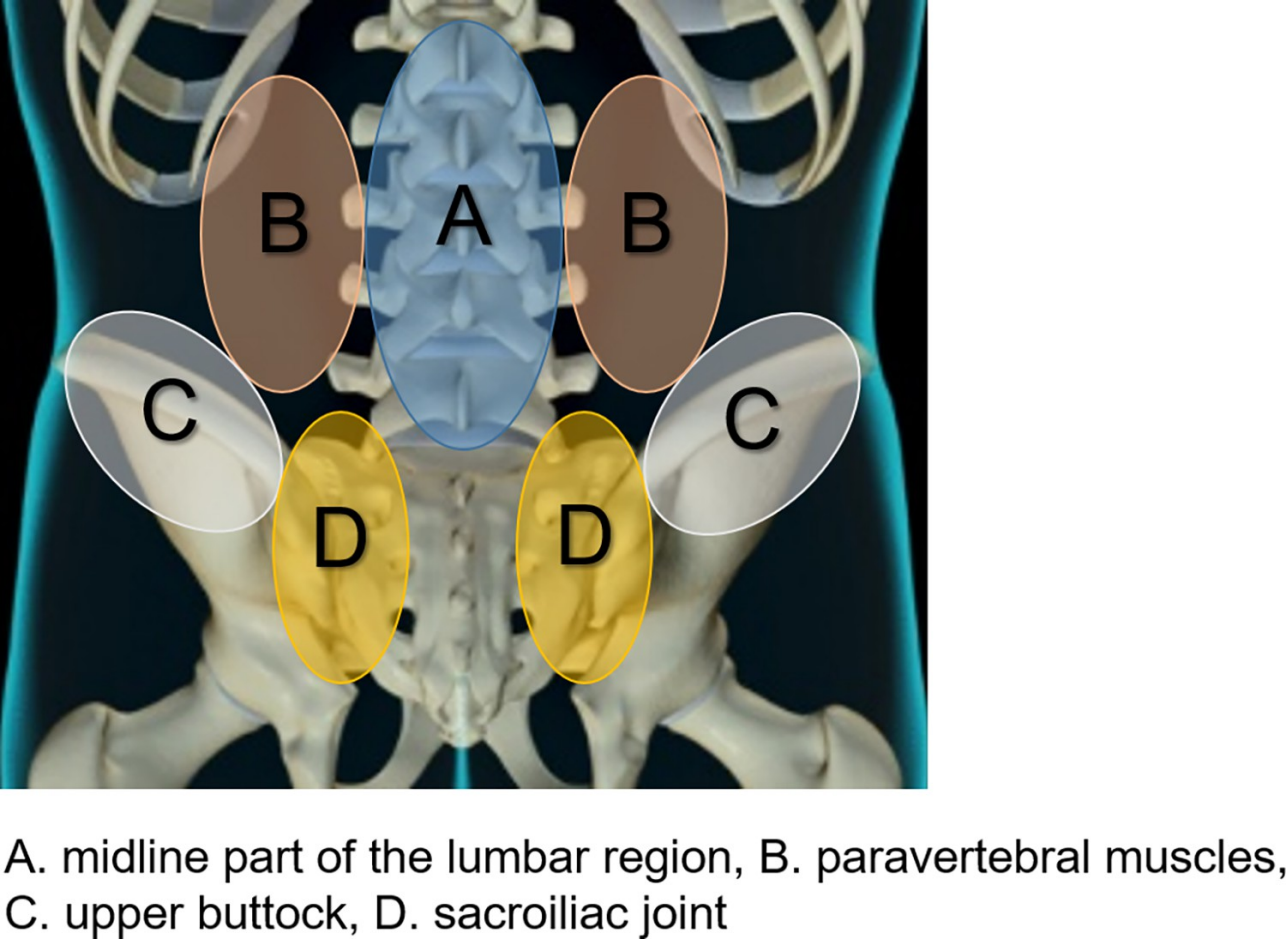
Localization diagnosis of low back pain. The location of low back pain (LBP) was categorized into four areas by palpation: A. midline of the lumbar region; B. paravertebral muscles; C. upper buttock; and D. sacroiliac joint.

After the clinical interview, the participants were in a prone position on an examination table. Experienced orthopedist (N.T.) performed physical examinations as previously reported with some modifications to assess the localization diagnosis of LBP [6]. The tender point of the lumbar area where the participants usually feel pain was determined by palpation. A rater performed posterior-anterior spring palpation by palm over each of the following four areas. The location of LBP was categorized into four areas: A. midline of the lumbar region, B. paravertebral muscles, C. upper buttock, and D. sacroiliac joint (Fig 1).

The midline of the lumbar region (A) is the median part of the spinal column. Paravertebral muscles (B) were those on the right and/or left sides of the spinal column where the erector spine muscles are located. The upper buttock (C) is the area around and immediately above the posterior iliac crest. The sacroiliac joint (D) is around the posterior-superior iliac spine (PSIS).

### Evaluation of QoL

LBP-related QoL was measured using the Oswestry Disability Index (ODI) [15]. The Japanese version of the ODI, the high reliability and validity of which has been reported [16], was used in this study. Health-related QoL was measured using the EuroQol 5 dimension (EQ-5D) that has two parts: EQ-5D self-classifier and EuroQol-visual analogue scales (EQ-VAS) [17].

### Statistical analysis

The association between the presence or location of LBP and health-related QOL scores was statistically evaluated using the Mann-Whitney U test or the Kruskal-Wallis test followed by post hoc multiple comparisons using the Dunn-Bonferroni method. The relationships between the location of LBP and NRS scores in several situations/positions were statistically analyzed using the Kruskal-Wallis test followed by post hoc multiple comparisons using the Dunn-Bonferroni method. Associate factors of the presence of LBP were identified by logistic regression analysis. Factors included in the logistic model were age, gender, body height, body weight, BMI, BMD, and presence of comorbidities (hypertension, diabetes mellitus, cerebrovascular disorder, ischemic heart disease, and smoking.) The variance inflation factor (VIF) was used to detect the degree of multicollinearity among the variables. VIF > 10 was considered indicative of multicollinearity and should be excluded from the regression model. There was multicollinearity among body height, body weight, and BMI (VIF >10). Therefore, body height and body weight were excluded from the analysis. All statistical analyses were performed using IBM SPSS Statistics. A P value <0.05 was considered significant.

## Results

### Participants’ characteristics

176 inhabitants received a medical examination of this study. Two inhabitants were excluded from this study because they had a history of lumbar spinal surgery. Therefore, 174 participants (53 men, 121 women; mean age: 72.3 years-old) were analyzed in this study. 93 participants (53.4% of total) who had experienced LBP in the past three months were included in the LBP-positive group. The characteristics of the total participants with or without LBP are summarized (Table 1). No inhabitants had comorbidities such as dementia, Parkinsonism, poorly controlled diabetes (glycated hemoglobin ≥7.0%), or acute vertebral fractures. There were no significant differences in age, gender, body height, body weight, BMI, BMD, and the prevalence of comorbidities between the LBP-negative and -positive groups. Logistic regression analysis did not show significant associate factors for the presence of LBP. ODI scores of the LBP-positive group was significantly higher than those of the LBP-negative group (P<0.01). EQ-5D, and EQ-VAS scores of the LBP-positive group were significantly lower than those of the LBP-negative group (P<0.01). 69 participants (74.2% of LBP-positive group) had only one location of LBP (LBP-one location group) (Table 1). 17 participants had two locations of LBP, 5 participants had three locations, and 2 participants had four locations.

**Table 1:** Characteristics of subjects with or without low back pain.

	Total subjects (n = 174)	LBP-negative (n = 81)	LBP-positive (n = 93)	P-value	LBP-one location (n = 69)	P-value (vs. LBP-negative)
**Men/Women**	53/121	29/52	24/69	n.s.	19/50	n.s.
**Age (years-old)**	72.3 (8.7)	72.1 (8.3)	72.4 (9.0)	n.s.	72.4 (9.2)	n.s.
**Body height (cm)**	154.6 (8.5)	155.7 (9.0)	153.6 (7.9)	n.s.	154.1 (7.9)	n.s.
**Body weight (kg)**	54.4 (9.8)	55.1 (10.0)	53.8 (9.6)	n.s.	54.6 (9.9)	n.s.
**BMI (kg/m** ^**2**^ **)**	22.7 (3.1)	22.7 (3.3)	22.7 (3.0)	n.s.	22.9 (3.2)	n.s.
**BMD**	82.5 (13.6)	83.6 (14.9)	81.6 (12.4)	n.s.	81.5 (12.0)	n.s.
**Hypertension**	53.4% (n = 93)	50.6% (n = 41)	55.9% (n = 52)	n.s.	55.1% (n = 38)	n.s.
**Diabetes mellitus**	10.3% (n = 18)	11.1% (n = 9)	9.7% (n = 9)	n.s.	8.7% (n = 6)	n.s.
**Cerebrovascular disorder**	3.4% (n = 6)	2.5% (n = 2)	4.3% (n = 4)	n.s.	5.8% (n = 4)	n.s.
**Ischemic heart disease**	6.9% (n = 12)	7.4% (n = 6)	6.5% (n = 6)	n.s.	5.8% (n = 4)	n.s.
**Smoking**	5.2% (n = 9)	6.2% (n = 5)	4.3% (n = 4)	n.s.	2.9% (n = 2)	n.s.
**ODI (%)**	11.2 (11.9)	7.3 (10.3)	14.6 (12.0)	<0.01	13.3 (11.2)	<0.01
**EQ-5D**	0.866 (0.189)	0.916 (0.147)	0.824 (0.210)	<0.01	0.836 (0.186)	<0.01
**EQ-VAS**	75.1 (14.5)	79.2 (13.8)	71.6 (14.1)	<0.01	72.3 (13.3)	<0.01

LBP: Low back pain; BMI: Body mass index; BMD: Bone mineral density; ODI: Oswestry Disability Index; EQ-5D: EuroQol-5 dimension; EQ-VAS: EuroQol-visual analogue scales. Number in parentheses indicates standard deviation (SD).

### LBP in several situations/positions

NRS scores of both the LBP-positive and LBP-one location groups were highest in the standing position, followed by at morning awakening and the sitting position; those scores were significantly higher than those of the LBP-negative groups (all, P<0.01) (Table 2). NRS scores in the lying position were significantly lower than those in the standing and sitting positions, and at morning awakening (total subjects and LBP one-location group: P<0.05; LBP-positive group: P<0.01) (Table 2).

**Table 2:** Characteristics of low back pain intensity.

	Total subjects (n = 174)	LBP-negative (n = 81)	LBP-positive (n = 93)	P-value	LBP one-location (n = 69)	P-value (vs. LBP-negative)
**Morning awakening**	1.1 (1.9)	0.2 (0.7)	2.0 (2.2)	<0.01	1.7 (2.1)	<0.01
**Walking**	0.9 (1.8)	0.2 (0.8)	1.5 (2.1)	<0.01	1.3 (2.0)	<0.01
**Standing**	1.5 (2.3)	0.6 (1.6)	2.3 (2.6)	<0.01	1.7 (2.2)	<0.01
**Sitting**	1.2 (1.9)	0.6 (1.4)	1.8 (2.1)	<0.01	1.5 (1.8)	<0.01
**Lying**	0.6 (1.5)*	0.3 (1.2)	0.8 (1.7)**	<0.05	0.8 (1.7)*	0.06

Numerical rating scale (NRS) scores in the lying position are significantly lower than those at morning awakening, while standing, and in the sitting position (

*P<0.05

** P<0.01). Number in parentheses indicates standard deviation (SD).

### Location of LBP

**Fig 2:**
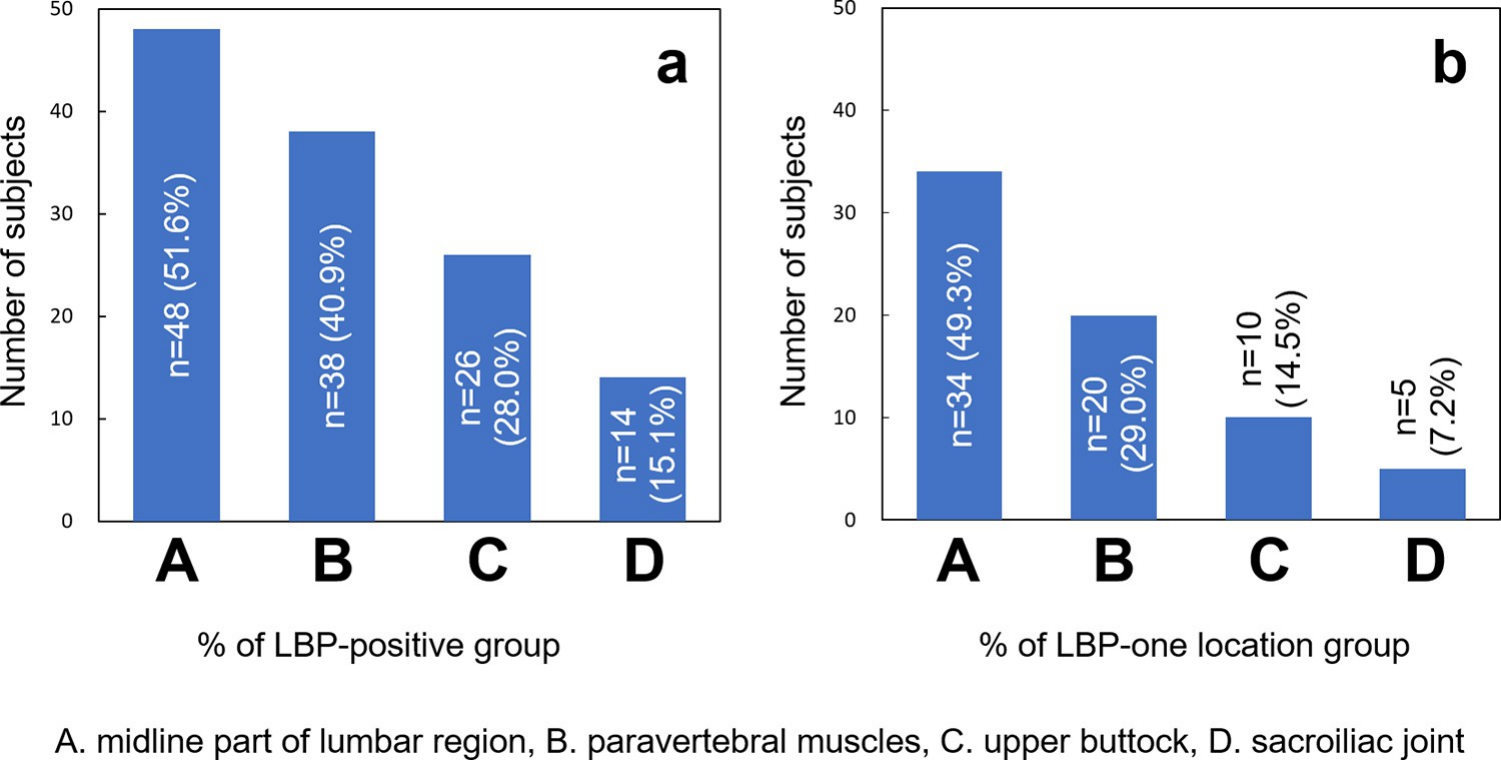
Location of low back pain. **a:** Number of subjects in LBP-positive group; **b:** Number of subjects in LBP-one location group. LBP: Low back pain; A. midline of the lumbar region, B. paravertebral muscles, C. upper buttock, and D. sacroiliac joint.

Among 93 participants who had LBP (LBP-positive group), 48 participants (51.6% of the LBP-positive group) had LBP at the midline of the lumbar region (A), 38 participants (40.9%) at the paravertebral muscles (B), 26 participants (28.0%) at the upper buttock (C) and 14 participants (15.1%) at the sacroiliac joint (D) (Fig 2A).

Among 69 participants who had LBP in one location (LBP-one location group), 34 participants (49.3% of LBP-one location group) had LBP at the midline of the lumbar region (A), 20 participants (29.0%) at the paravertebral muscles (B), 10 participants (14.5%) at the upper buttock (C) and 5 participants (7.2%) at the sacroiliac joint (D) (Fig 2B). There were no significant differences in age, gender, body height, body weight, BMI, BMD, and prevalence of comorbidities among the location of LBP (Table 3).

**Table 3:** Characteristics of subjects who had LBP in one location (LBP-one location group).

	A (n = 34)	B (n = 20)	C (n = 10)	D (n = 5)	P-value
**Men/Women**	13/21	2/18	2/8	2/3	n.s.
**Age (years-old)**	73.6 ± 8.8	70.4 ± 9.6	73.9 ± 10.6	68.8 ± 8.5	n.s.
**Body height (cm)**	154.5 ± 8.4	153.0 ± 7.0	153.7 ± 9.3	156.7 ± 7.0	n.s.
**Body weight (kg)**	54.8 ± 10.0	52.5 ± 9.7	58.3 ± 12.1	53.9 ± 6.3	n.s.
**BMI (kg/m** ^**2**^ **)**	22.9 ± 3.3	22.4 ± 3.2	24.6 ± 3.5	21.9 ± 1.6	n.s.
**BMD**	80.5 ± 12.4	80.9 ± 11.9	82.9 ± 13.8	87.8 ± 9.5	n.s.
**Hypertension**	64.7% (n = 22)	40.0% (n = 8)	50.0% (n = 5)	60.0% (n = 3)	n.s.
**Diabetes mellitus**	11.8% (n = 4)	5.0% (n = 1)	5.0% (n = 1)	0% (n = 0)	n.s.
**Cerebrovascular disorder**	11.8% (n = 4)	0% (n = 0)	0% (n = 0)	0% (n = 0)	n.s.
**Ischemic heart disease**	11.8% (n = 4)	0% (n = 0)	0% (n = 0)	0% (n = 0)	n.s
**Smoking**	2.9% (n = 1)	0% (n = 0)	10.0% (n = 1)	0% (n = 0)	n.s

LBP: Low back pain; BMI: Body mass index; BMD: Bone mineral density.

### Relationship between the intensity of low back pain in different situations/positions and the location of low back pain

**Fig 3:**
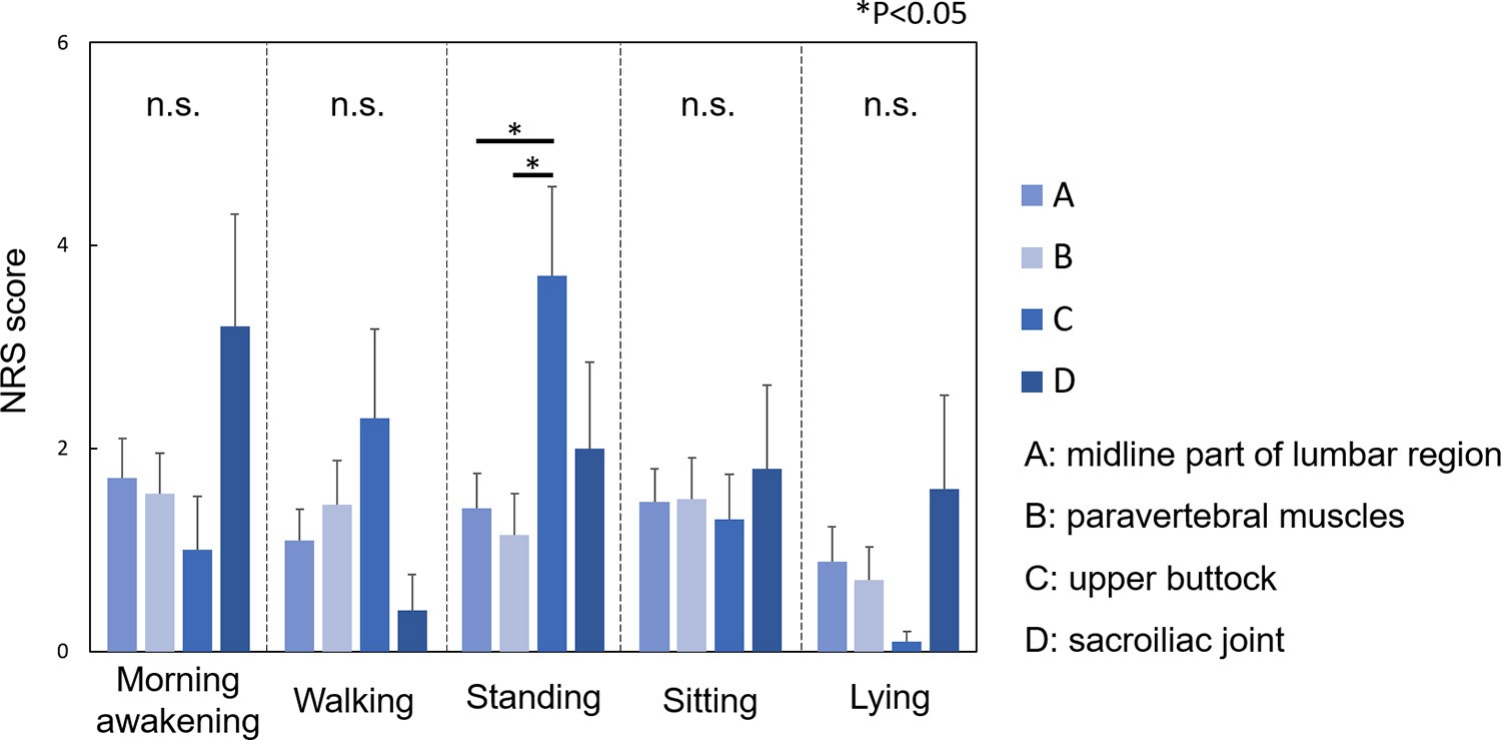
Relationship between the intensity of low back pain and the location of low back pain. NRS scores of the LBP-one location group in several situations/positions were assessed by the location of LBP. 1. Morning awakening, 2. Walking, 3. Standing, 4. Sitting, and 5. Lying down. A. midline of the lumbar region, B. paravertebral muscles, C. upper buttock, and D. sacroiliac joint. NRS: Numerical rating scale; LBP: Low back pain.

The intensity of LBP in several situations/positions was evaluated at each location of LBP (Fig 3). In the standing position, NRS score of LBP at the upper buttock (C) was significantly higher than those of LBP at the midline of the lumbar region (A) and the paravertebral muscles (B) (P<0.05). However, there were no significant differences in the NRS scores among the location of LBP in morning awakening, walking, sitting, and lying.

**Fig 4:**
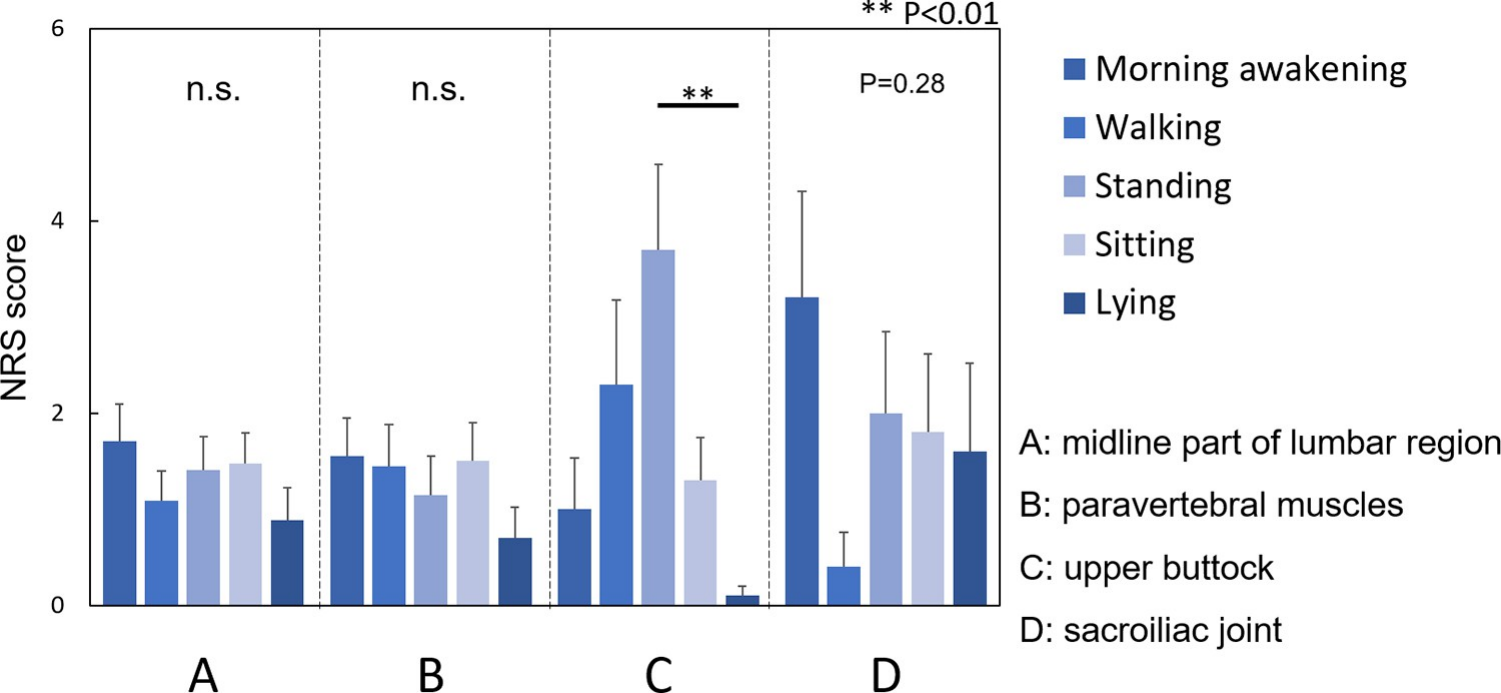
Relationship between the intensity of low back pain in different situations/positions and the location of low back pain. The differences in NRS scores of the LBP-one location group were evaluated in different situations/positions. 1. Morning awakening, 2. Walking, 3. Standing, 4. Sitting, and 5. Lying down. A. midline of the lumbar region, B. paravertebral muscles, C. upper buttock, and D. sacroiliac joint. NRS: Numerical rating scale; LBP: Low back pain.

The differences in NRS scores for LBP were evaluated in different situations/positions. Among the participants who had LBP at the upper buttock (C), NRS scores in the standing position were significantly higher those in the lying position (P<0.01) (Fig 4). Among the participants who had LBP at the sacroiliac joint (D), NRS scores at morning awakening were the highest, and those while walking were the lowest; however, these differences did not reach statistical significance (P = 0.28, Fig 4).

### Relationship between the location of LBP and the extent of QoL issues

**Fig 5:**
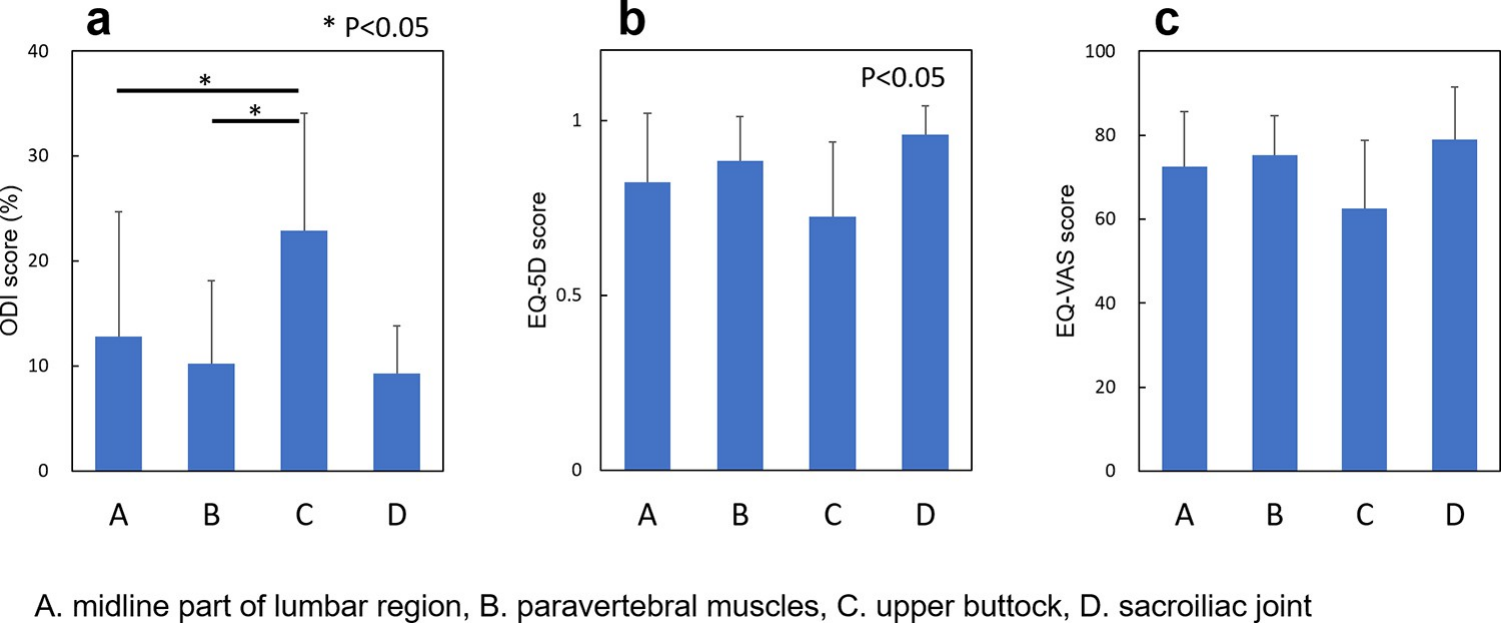
Relationship between the location of low back pain and the extent of QoL issues. **a:** ODI scores of the LBP-one location group; **b:** EQ-5D scores of the LBP-one location group; **c:** EQ-VAS score of LBP-one location group; A. midline of the lumbar region, B. paravertebral muscles, C. upper buttock, D. sacroiliac joint. LBP: Low back pain; QoL: Quality of life; ODI: Oswestry Disability Index; EQ-5D: EuroQol-5 dimension, EQ-VAS: EuroQol-visual analogue scale.

The relationship between the location of LBP and the extent of QoL issues was assessed among the participants who had LBP in one location. The ODI scores of participants who had pain at the upper buttock (C) was significantly higher than those of participants who had pain at the midline of the lumbar region or paravertebral muscles (P<0.05, respectively) (Fig 5A). The EQ-5D score of participants differed significantly depending on the LBP location (P<0.05) (Fig 5B). There was no significant difference in EQ-VAS scores among the locations of LBP (P = 0.17) (Fig 5C).

## Discussion

There has been only one previous epidemiological study that evaluated the anatomic location of LBP. Thiese et al. evaluated the location of LBP of 828 workers in a cross-sectional study [18]. They reported that 44.0% of participants had LBP within the past month, and the most common area of LBP was in the immediate paraspinal area (33.6% of total participants); this was similar to the results of our study.

LBP at the midline of the lumbar region (area A in our study) was reported as ‘the presence of pain on firm palpation of the lumbar spinous process in the middle’ [6,7]. Schneider et al. performed a palpation method for 39 patients with LBP and evaluated the interrater reliability [6]. Similar to our method, the participants were in prone on an examination table. Then, a rater performed posterior-anterior spring palpation using a palm over the lumbar spinous process in the middle to evaluate a pain provocation. They reported that adjusted kappa values for the lumbar spinous process in the middle were 0.34 (L1 to L3) and 0.58 (L4 and L5).

LBP at the midline of the lumbar region (area A) may include symptoms caused by discogenic pain, lumbar spinal stenosis, or lumbar spondylosis. There is growing evidence that lumbar disc degeneration is one of the major causes of LBP [19]. Fujii et al. described diagnostic methods for discogenic pain in a literature review [20]. To diagnose discogenic pain, the advantage of provocative discography is its relatively high specificity and sensitivity. However, discography may be too invasive to use as a diagnostic procedure. MRI findings, including type 1 Modic changes and high intensity zones, seem especially useful. However, at present, the specificity and/or sensitivity on MRI findings are not sufficient and show only moderate effectiveness in fully identifying a source of pain.

In a retrospective chart review, Depalma et al. aimed to estimate the sensitivity, specificity, positive and negative predictive values, diagnostic accuracy, and likelihood ratios of positive and negative tests for diagnosing internal disc disruption (IDD), facet joint pain (FJP) or sacroiliac joint pain (SIJP) by use of the presence of midline and paramidline LBP. During the clinical evaluation, patients pointed to the most painful LBP with one finger, which was documented as midline (by the spinous processes) or paramidline (more than one fingerbreadth lateral to the midline). In cases of IDD, significantly greater percentages of patients reported midline LBP compared to FJP or SIJP and significantly lower percentages of patients reported paramidline pain compared to FJP or SIJP. Depalma et al. concluded that a spine specialist could predict the source of a patient’s LBP by evaluating the location of LBP because the presence of midline LBP increases the probability of lumbar IDD [21].

On the other hand, Allegri et al. reported that one of the most frequent symptoms of lumbar stenosis was midline back pain [22]. Therefore, it is possible that pain at the midline of the lumbar region (area A) would anatomically originate from a lumbar intervertebral disc lesion, but the neuropathic origin of the pain by canal stenosis should also be included.

Suzuki et al. reported that among 320 outpatients with LBP, 68 patients (21%) had FJP, and 56 patients (18%) had fascial LBP [2]. They also evaluated sensitivity and specificity of one point tenderness at the paravertebral muscles for patients with FJP and fascial LBP (FJP: sensitivity: 0.706; specificity: 0.631; fascial LBP: sensitivity: 0.696; specificity: 0.614). Therefore, LBP at the paravertebral muscles (area B) may indicate symptoms from FJP or fascial LBP. Similar to our methods, Schneider et al. performed posterior-anterior spring palpation using a palm over the lumbar facet joint and showed that adjusted kappa values for palpation of FJP were 0.48 (L1/L2 to L3/L4 facets) and 0.74 (L4/L5 and L5/S1 facets) [6].

Superior cluneal nerve entrapment neuropathy (SCN-EN) is one of the causes of LBP at the upper buttock (area C) [23,24]. In 1957, Strong and Davila reported that the SCN can be entrapped around the iliac crest [25]. The incidence of SCN-EN in patients with LBP was reported to be 1.6%–14% [24,26].

In our study, 26 participants (28.0% of the LBP-positive group) had LBP at the upper buttock; its prevalence was higher than previously reported. Differences in ages of the subjects and/or diagnosis methods could have contributed to the discordance in prevalence between our and previously reported data.

In our study, ODI scores [15] of participants who had pain at the upper buttock were significantly higher, and the EQ-5D scores were significantly lower than those who had pain at other locations. It has been reported that LBP caused by SCN entrapment is induced and exacerbated by movements such as prolonged sitting, rising from seated position, standing, or walking [23]. Our study also showed that NRS scores of participants who had LBP at the upper buttock in the standing position were the highest, followed by while walking. Therefore, our study suggests that pain at the upper buttock during these movements is significantly associated with exacerbation in intensity of pain and health related QOL.

Pain at/around the PSIS (area D) is characteristic of sacroiliac joint (SIJ) -related pain. The prevalence of SIJ-related pain was reported at around 25% (range between 10% and 62%) in patients with LBP [27]. In accordance with previous study, our study showed that 15.1% of the LBP-positive group had pain at the posterior superior iliac spine. Murakami et al. reported that when patients can point to the PSIS or within 2 cm of it as the main site of pain with the one-finger test, the SIJ should be considered as the origin of the LBP [28]. The interrater reliability of the pain provocation test for SIJ was also reported as excellent reliability values (kappa coefficient = 0.81) [7]. Like Murakami’s study, the tender point of the lumbar area was determined by palpation to assess a localization diagnosis of LBP in our study.

Aoki et al. reported that one hundred eighty-nine patients with nonspecific LBP were evaluated for the extent of LBP in three different postural situations (in motion, standing, and sitting) [29]. They reported that elderly patients showed significantly lower visual analogue scale (VAS) scores while sitting compared to young patients. However, no significant differences were observed in traditional VAS, VAS while in motion/standing, and ODI scores between the two groups.

We evaluated LBP of inhabitants in a greater number of different situations/positions and investigated their relationship with the location of LBP. Our study showed the extents of LBP at the upper buttock in the standing position and at the sacroiliac joint in morning awakening were higher.

It has been considered that LBP at the midline of the lumbar region or the paravertebral muscles was attributable to lumbar degenerative diseases. However, the pathogenesis of LBP at the upper buttock or the sacroiliac joint might include categories other than the lumbar spine, such as the pelvis. The localization diagnosis by palpation may help to categorize the pathogenesis of LBP into the lumbar spine or other locations in the clinical setting. If the pain is located in the median part or the right and/or left sides of the spinal column, imaging diagnosis such as radiography, MRI, or CT would be recommended to diagnose the lumbar degenerative diseases, including degenerative disc disease, spinal stenosis, or degenerative spondylosis. On the other hand, if the pain is located in the upper buttock or the sacroiliac joint, it would be necessary to distinguish the origin of pain from lumbar degenerative diseases. Additional physical examinations that reflect sacroiliac joint pain [28] would be recommended. Namely, the localization diagnosis was important as the first step in evaluating for the cause of LBP.

In the clinical setting, a clinician should perform physical examinations on all areas of the low back to identify the location of LBP by the palpation method. This examination can diagnose whether the LBP is caused by the lumbar spine or other sites, such as the pelvis. In addition, the imaging examinations such as radiographs, MRI, or CT are recommended. The localization diagnosis of LBP is a preferable method to increase the accuracy of identifying the pathogenesis of ‎LBP.

The present study has some limitations. First, Miyagawa (Odai-cho) is a typical mountain village, and many inhabitants are engaged in farming or forestry. Therefore, there would be differences in lifestyle compared with that of the general Japanese population. Secondly, participants in this study would be relatively healthy without severe physical disability, such as severe LBP. Third, BMD was measured on the forearm, not the lumbar spine. Fourth, one orthopedist performed physical examinations in our study; therefore, the interrater reliability of our palpation method was not assessed. A quantitative assessment of pressure during palpation has not been performed. Therefore, the same pressure may not be applied for the low back area of all participants. Imaging examinations or block injections of local anesthesia were not performed. Therefore, the definitive diagnosis was unknown. The evaluation of LBP location by palpation methods was not compared to the image evaluation by lumbar radiograph, CT, or MRI. Lastly, the lumbar location was not distinguishable between right and left sides. There was also a mixture of unilateral and bilateral pain in our study.

## Conclusions

Our study was the first report to investigate the location of LBP in a population-based study and to evaluate its association with QoL. The location of LBP could be successfully categorized by palpation into four areas. The localization of LBP was significantly associated with LBP intensity and LBP-related QoL.

## Supporting information

S1 Table LBP location, QOL scores, comorbidities. (XLSX)

S2 Table NRS score in situations/positions. (XLSX)

S3 Table NRS score in LBP location and situations/positions. (XLSX)
